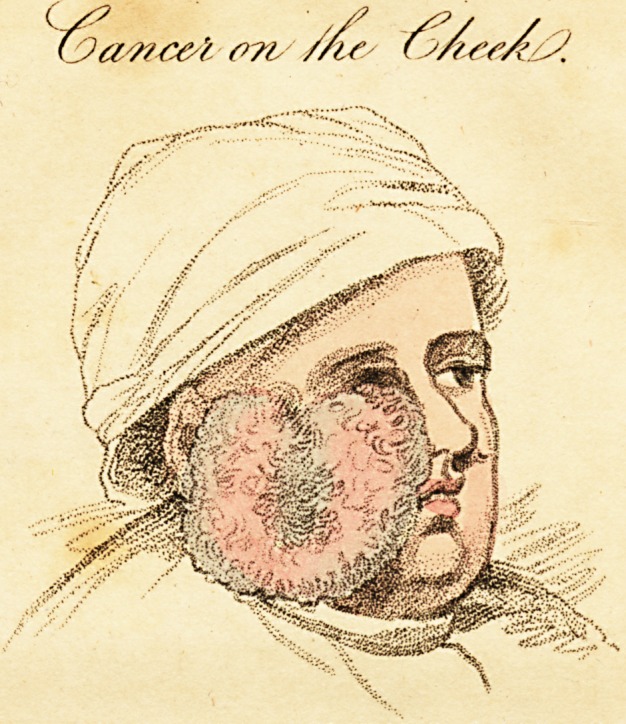# Mr. Atkinson, on Cancer

**Published:** 1801-11

**Authors:** James Atkinson

**Affiliations:** No. 8, Duncan Terrace, Islington


### *To the Editors of the Medical and Phyfical Journal.*

GENTLEMEN,

IT is to be lamented that the Science of Medicine fhould ever be difgraced by arrogant pretenders, or by thofe who, having difcoved any falutary mode of treating difeafes, withhold it from the world from bafe, narrow, and ungenerous motives. The man poffeffed of any important difcovery may do good to the perhaps contracted circle in which he practifes, while the reft of mankind are denied thofe benefits. It would be unreafonable to think, that after his affiduity, perplexity, and anxious enquiry in attaining fuch a defideratum, he fhould receive no reward. The ftruggles of genius ought always to meet with an ample compenfation, and every exertion for the general good duly appreciated.

Cancerous affections are truly dreadful, when proper remedies have not been applied in due time; they have baffled every exertion of medicine and of furgery, the patient lingering in torment for a length of years. Being in the neighbourhood of Rochefter, I was induced to fee a gentleman (Mr. Knight, of Friendfbury) who had a cancer on his right cheek. The tumour is very large, and has rendered the right eye nearly blind, extending to below the jaw, and from the nofe to the ear. The dreffings being removed, the tumour was of a florid red, and a quantity of blood oozed from it. The

The hiftory of the cafe is fhort: For four years it remained a fmall hard fpeck, about the fize of a pea, till fix months ago, when it daily increafed. The practitioner who then attended him, almoft choaking him with medicines to no good effect, at length ventured to ufe the knife. What was his intention, or what was then cut away, I could not learn; but it appears from that time the growth of the tumour was greatly accelerated. The apothecary who now attends him, equally deftitute of the talents and difcrimination requifite to form the able furgeon, prefcribes with unparalelled prefumption and effrontery, in a cafe, the treatment of which he is totally ignorant of. It is unfortunate that fuch fhocking difeafes fhould fall within the practice of fuch incompetent individuals, who are allowed to fport with the health of their fellow creatures. Mr. Knight, I believe, is between fifty and fixty years of age; he is in much pain; the tumour feems not to have eroded the infide of his mouth, as he feels no difagreeable tafte, which he muft have done had it been fo, from the acrimony and fetor of the difcharge. He gets little reft, and has terrifying dreams. Humanity, and a confcioufnefs of the cafe being at prefent injudicioufly treated, were the only motives for my fubmitting this paper to the judgement of your correfpondents, who may probably fuggeft fome means of alleviating his mifery. I have fubjoined a correct drawing *(see the plate)* of the fize and appearance of the tumour. The fmall cancerous fpot on his left cheek has been there two years.

I am, &c.

*No.* 8, *Duncan Terrace, Iflington.*

JAMES ATKINSON.

## Figures and Tables

**Figure f1:**